# The hidden diversity of ribosomal peptide natural products

**DOI:** 10.1186/1741-7007-8-83

**Published:** 2010-06-17

**Authors:** Eric W Schmidt

**Affiliations:** 1 Department of Medicinal Chemistry and Department of Biology, University of Utah, Salt Lake City, UT 84112, USA

## Abstract

A recent report in *BMC Biology *on the discovery and analysis of biosynthetic genes for ribosomal peptide natural products confirms that these pathways are much more common and diverse than previously suspected, contributing substantially to the chemical arsenal employed by bacteria.

See research article http://www.biomedcentral.com/1741-7007/8/70

## Commentary

Bacterial natural products such as nonribosomally synthesized peptides and polyketides are widely appreciated as biologically active agents, in part because they include many pharmaceuticals. Because their synthesis is encoded by large, highly conserved genes, their presence, distribution and abundance is easily assessed. By contrast, although the study of biologically active ribosomally synthesized peptides - ribosomal peptide natural products - has a long and rich history, the true structural diversity of these compounds is just beginning to be appreciated. In a recent paper in *BMC Biology*, Haft, Basu and Mitchell [1] report on informatics methods to uncover cryptic diversity in ribosomal peptide natural products, and in the process discover two widely occurring new families. Within these families, there are numerous precursor peptides with hypervariable sequences. These precursors can likely act as substrates for the processing pathways, and so each pathway leads to numerous different peptides. This work and other recent studies suggest that the ribosomal peptide natural products represent a largely hidden arsenal of active small molecules that are important in microbiology, the environment, medicine and technology.

## Synthesis of ribosomal peptide natural products

**Figure 1:**
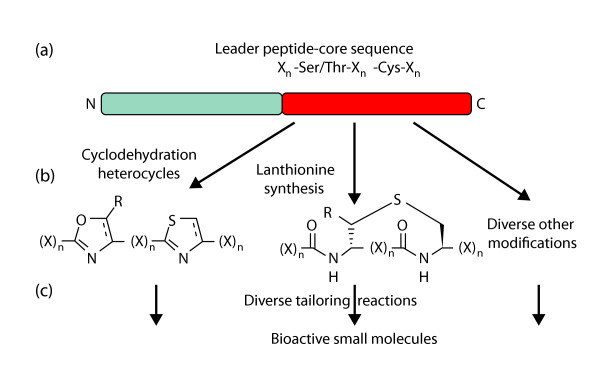
**Diversity in ribosomal peptide natural products is produced by variety in enzymatic modification and precursor peptide hypervariability**. **(a) **Although precursor peptides consist of many different apparently unrelated sequence groups, in general they contain a relatively conserved leader sequence directing enzyme modification (grey) and a hypervariable core sequence that encodes the final natural product (red). **(b) **In this study [1] cyclodehydrating and lanthionine-bond forming enzymes were analyzed, but other modifying enzymes exist, leading to numerous posttranslational modifications. **(c) **Further derivatization by multiple types of enzymes, including proteases that cleave the peptide product from the leader peptide, add to the chemical diversity of ribosomal peptide natural products.

Ribosomal peptide natural products are derived from short precursor peptides, most commonly around 100 amino acids long, that are posttranslationally modified by various enzymes that catalyze the formation of a large number of different chemical motifs (Figure 1) [2,3]. Commonly, the precursor peptide contains a relatively conserved leader sequence that is at least partly responsible for recognition by the modifying enzymes and/or by export machinery such as ABC transporters. The carboxyl terminus of the precursor peptide encodes the sequence that is enzyme-modified (the 'core sequence'). Usually, the leader is cleaved from the mature carboxyl terminus following modification, resulting in a short peptide product. Mutations in the carboxy-terminal core sequence are often tolerated by modifying enzymes, leading to natural libraries of peptide products [4]. These biosynthetic trends are nearly universal for the bacterial ribosomal peptide natural products and are also commonly found in the biosynthesis of similar peptides from other organisms such as cone snails and fungi.

Despite these biosynthetic relationships, enzymes and precursor peptides are often not clearly homologous. In addition, the bacterial ribosomal peptide literature is fragmented. These natural products were originally described as 'bacteriocins', peptides that inhibited the growth of bacteria closely related to the producing strain. Low-molecular-weight bacteriocins include the lantibiotics and the microcins, which are highly posttranslationally modified peptides that in some ways resemble their nonribosomally synthesized cousins ([2] and references therein). A major problem with this nomenclature, however, is that many bacteriocins are proteins unrelated to the small-molecule bacteriocins. In addition, many of the ribosomal peptides exhibit strikingly different activities from those conventionally ascribed to bacteriocins, including roles in the induction of genetic competence, quorum sensing and enzyme catalysis (as small-molecule redox cofactors). Some peptides show *in vitro *activity against mammalian cells but not against bacteria or fungi, and the biological function of many others is unknown. The enormous number of structural classes and of molecules within each class suggests that these small peptides have many different biological roles that have yet to be investigated. The report by Haft *et al*. [1] directly demonstrates the complexity and interrelatedness of the lantibiotic and microcin groups and will help in the push to consolidate the literature and to understand the biological roles of these ubiquitous compounds.

## Making connections among the heterocyclic natural products

The genes for ribosomal peptide natural products are hard to find, especially in the absence of any chemical or bioactivity information. The precursor peptides are small and often only distantly related to other precursor peptides, and so are often not called as coding sequences in automatic genome annotation [5]. Similarly, new families of modifying enzymes are often not closely enough related to characterized relatives to be identified by BLAST searching [6,7]. A particularly revealing story involves cyclodehydratase-mediated posttranslational modification of cysteine, serine and threonine residues to form heterocyclic thiazole and oxazole moieties. For the antibacterial ribosomal peptide microcin B17 from *Escherichia coli*, Walsh and colleagues [8] showed that a three-protein enzyme complex, including cyclodehydratase, was required to modify the precursor peptide. A study by our group [6] on the patellamides, small heterocyclic ribosomal peptides from cyanobacterial symbionts of marine animals, showed that the biosynthetic pathway did not contain a cyclodehydratase or a precursor peptide with significant sequence identity to microcin B17, despite identical heterocyclization biochemistry. Instead, a single-subunit cyclodehydratase was identified. Homologous enzymes have been recognized in the genomes of the cyanobacterium *Trichodesmium erythraeum *(leading to the discovery of a new peptide product [5,6]) and *Streptococcus iniae *(leading to the recognition of streptolysin S as a thiazole-containing product) [6,7].

Using an informatics approach, Dixon and colleagues [7] showed that the cyclodehydratases that modify microcin B17, patellamides and other classes of peptides are extremely abundant in bacteria and are linked to diverse other posttranslational modifications. Despite extreme sequence dissimilarity among individual members, informatics studies revealed previously cryptic homology in the cyclodehydratases. Recently, the importance of cyclodehydratases in the production of therapeutics has been emphasized by the independent discovery by several groups that the thiostrepton family of antibiotics is also ribosomally synthesized [9].

The study by Haft *et al*. [1] significantly extends this work. The authors searched for gene clusters involved in the biosynthesis of what they refer to as thiazole/oxazole-modified microcins (TOMMs) in more than 1,000 available bacterial genome sequences, specifically searching for cyclodehydratase protein sequences. Two new precursor peptide classes were discovered, both of which are related to larger proteins. One is related to a non-catalytic fragment of nitrile hydrolase (NHase) and the other to the Nif11 proteins involved in nitrogen fixation. Strikingly, in some genomes the NHase-like precursor peptide gene clustered with the cyclodehydratase gene, whereas in other genomes it was located far away or the two genes were even separated on a plasmid/chromosome pair. Further careful analysis using recently developed informatics tools has enabled identification of a transport protein linked to this system [1]. The NHase-like group of precursor peptides was found in phylogenetically diverse bacteria, indicating a potentially broadly important new class of secondary metabolites.

The Nif11-related precursor peptides are particularly interesting because of the potential relationship to nitrogen fixation. While some Nif11-like precursor genes were linked to cyclodehydratase genes, others were linked to genes encoding the export machinery. In a striking twist, some of the Nif11-like precursor genes were linked to genes for enzymes that carry out a completely different posttranslational modification that leads to a lantibiotic. This result clearly shows that microcins and lantibiotics, while superficially seeming structurally and biochemically dissimilar, are actually closely linked in their precursors and biosynthesis. Haft *et al*. [1] suggest that further studies of this type will help to uncover numerous TOMM (and other natural product) orphan genes. The new informatics methods and ideas developed by the authors will greatly help to further define the evolutionary routes and interrelationships in the ribosomal peptide group.

The functionality of the TOMM genes was demonstrated recently by van der Donk and colleagues [10], who found Nif11-like precursor peptides and their lantibiotic-pathway processing machinery in strains of the ubiquitous planktonic marine cyanobacterium *Prochlorococcus*. The *Prochlorococcus *genus is responsible for a large percentage of global carbon fixation and is found in tropical and subtropical oceans around the world. Previously, no natural products had been isolated from members of this genus and their genomes are quite small and would traditionally be considered unlikely to encode many new compounds. However, about 0.5 to 5% of strains of *Prochlorococcus *and the related genus *Synechococcus *in environmental databases are estimated to encode lantibiotic synthesis [10]. A single strain, *Prochlorococcus *MIT9313, contained 29 precursor peptides that could be modified by a single lantibiotic-processing enzyme, leading to a natural library of diverse natural products in these strains. The paralogous expansion of precursor peptides in *Prochlorococcus *and other species was also recognized by Haft *et al*. [1]. Altogether, these studies indicate that the widespread TOMM group identified by Haft *et al*. does indeed lead to predicted new natural products in important bacterial strains. The biological role of such peptides remains speculative.

## Precursor peptides and natural product diversity

By necessity, an enzyme that is capable of modifying 29 diverse precursor peptides must exhibit broad substrate tolerance. Indeed, work by numerous groups has established that ribosomal-peptide-modifying enzymes often accept numerous substitutions in the core sequences of precursor peptides, as long as the leader sequences are somewhat conserved. This feature has allowed both *in vivo *and *in vitro *synthesis of diverse ribosomal peptide derivatives.

The biological impact of this broad substrate specificity has been studied in our lab using the patellamide pathway [4]. We examined symbiotic cyanobacteria in 46 different marine animals from across the tropical Pacific and showed that the DNA encoding modifying enzymes and leader sequences were identical, within PCR error, in these closely related symbiotic strains. However, the core sequences were hypervariable, encoding a small library of 29 different precursor peptides. This population of so-far uncultured symbiotic cyanobacteria, which are present in numerous marine animals, synthesizes an enormous diversity of natural products using identical enzymes encoded by identical genes.

The *Prochlorococcus *paper [10] and the work of Haft *et al*. [1], along with other recent advances cited therein, reveal that hypervariability in core sequences is a general phenomenon with a widespread impact. To the best of my knowledge, the presence of identical (at the DNA sequence level) enzymes and leader sequences with hypervariable core sequences in different strains has not yet been described beyond the symbiotic *Prochloron *studied in our lab. Nonetheless, strikingly similar stories are clearly apparent in the sequence alignments obtained by Haft *et al*. [1] and previous reports. Numerous variations in modifying enzymes have been discovered previously and the numbers of known enzymes are expanding. As many of these enzymes apparently synthesize diverse products using hypervariable precursor peptides, it is clear that the true structural diversity and biological impact of the ribosomal peptide natural products is just beginning to be appreciated. The informatics approach reported by Haft *et al*. will be very useful in the quest for new ribosomal peptides.

## Remaining fundamental questions

The 'bacteriocins' have been studied for nearly 90 years, yet key questions of broad importance to their biology have yet to be addressed. The evolutionary relationships between diverse peptide families have yet to be established. More fundamentally, the evolution of hypervariable core sequences within very highly conserved DNA sequence backgrounds has not been examined. How do bacteria produce extreme variation in small cassettes of around 9 to 60 nucleotides while the remainder of the DNA remains constant? This could be related to known phenomena such as pilin variation, but this has yet to be established. As such core variation is clearly a general phenomenon in bacteria, it would be useful to determine its mechanism. Finally, although the roles of some groups of bacteriocins have been extensively studied and mathematically modeled, we do not yet understand the biological roles of most of them. In developing methods to find and analyze precursor and enzyme relationships, the work of Haft and co-workers will greatly aid studies to answer these fundamental questions.
